# Biocuration of a Transcription Factors Network Involved in Submergence Tolerance during Seed Germination and Coleoptile Elongation in Rice (*Oryza sativa*)

**DOI:** 10.3390/plants12112146

**Published:** 2023-05-29

**Authors:** Sushma Naithani, Bijayalaxmi Mohanty, Justin Elser, Peter D’Eustachio, Pankaj Jaiswal

**Affiliations:** 1 Department of Botany and Plant Pathology, Oregon State University, Corvallis, OR 97331, USA; justin.elser@oregonstate.edu (J.E.); pankaj.jaiswal@oregonstate.edu (P.J.); 2 NUS Environmental Research Institute, National University of Singapore, Singapore 117411, Singapore; bijayalaxmi.mohanty@gmail.com; 3 Department of Biochemistry and Molecular Pharmacology, NYU Grossman School of Medicine, New York, NY 10016, USA

**Keywords:** rice, plant pathways modeling, gene biocuration, Plant Reactome, Gramene, submergence stress, transcription factor, seed germination, coleoptile elongation, gravitropism

## Abstract

Modeling biological processes and genetic-regulatory networks using in silico approaches provides a valuable framework for understanding how genes and associated allelic and genotypic differences result in specific traits. Submergence tolerance is a significant agronomic trait in rice; however, the gene–gene interactions linked with this polygenic trait remain largely unknown. In this study, we constructed a network of 57 transcription factors involved in seed germination and coleoptile elongation under submergence. The gene–gene interactions were based on the co-expression profiles of genes and the presence of transcription factor binding sites in the promoter region of target genes. We also incorporated published experimental evidence, wherever available, to support gene–gene, gene–protein, and protein–protein interactions. The co-expression data were obtained by re-analyzing publicly available transcriptome data from rice. Notably, this network includes OSH1, OSH15, OSH71, Sub1B, ERFs, WRKYs, NACs, ZFP36, TCPs, etc., which play key regulatory roles in seed germination, coleoptile elongation and submergence response, and mediate gravitropic signaling by regulating OsLAZY1 and/or IL2. The network of transcription factors was manually biocurated and submitted to the Plant Reactome Knowledgebase to make it publicly accessible. We expect this work will facilitate the re-analysis/re-use of OMICs data and aid genomics research to accelerate crop improvement.

## 1. Introduction

Rice (*Oryza sativa L*.) is one of the world’s major staple crops. It is unique among cereals in that its seeds can germinate under submergence [1,2,3,4,5,6]. Submergence tolerance in rice at an early stage of seed germination and coleoptile elongation is an important agronomic trait. It allows direct seeding methods in rainfed and flood-affected areas and, thus, helps reduce labor costs associated with conventional rice transplanting [7,8]. However, rice genotypes differ in their submergence tolerance, and most rice varieties show less than 50% survival at the early seedling stage under submergence [9]. When submerged in water, germinating rice seeds encounter low oxygen conditions (hypoxia or anoxia) that trigger coleoptile emergence [7]. The carbohydrate reserve of the seed supports coleoptile elongation. The coleoptile protects the true leaves, provides nutrients for the developing tissues, and accesses oxygen to support the seedling establishment [10]. Under anaerobic conditions, the coleoptile elongates to reach the water’s surface for oxygen diffusion, which is critical for initiating root and shoot development [8,10]. Compared with sensitive rice genotypes, submergence-tolerant genotypes exhibit substantial coleoptile elongation due to their ability to use stored starch reserves efficiently (higher amylase activity), lower peroxidase activity, and higher ethylene production in germinating seeds—all of which increase seedling survival [11,12,13,14]. The submergence tolerance at the early stage of seed germination and seedling growth is also important for the plants grown in microgravity environments on spaceflights and at space stations. In particular, the absence of convection-driven gas movement in the environment of spaceflights and space stations leads to reduced bioavailability of oxygen in imbibed seeds, which can cause hypoxia stress [15,16]. This stress condition is similar to that induced by submergence. Therefore, it is essential to have a fundamental understanding of genes, gene–gene interactions, and the mechanisms that facilitate seed germination under submergence and hypoxia stress. This knowledge is crucial not only for enhancing direct seeding practices in paddy fields but also for advancing the development of sustainable bioregenerative life support systems for astronauts on long-term space flights and the International Space Station (ISS), and for the potential colonization of the Moon and Mars [17].

The rate of seed germination under submergence is controlled by the synergistic action of phytohormones, including ethylene, gibberellic acid (GA), abscisic acid (ABA), auxins, jasmonic acid (JA), brassinosteroids (BR) and salicylic acid (SA) (for a recent review, see [8,18]). Phytohormones play a crucial role in integrating intrinsic developmental and extrinsic gravitropic signals to regulate seed germination and coleoptile growth [19]. Among the various phytohormones, ethylene plays a crucial role as a primary signaling molecule in regulating responses to hypoxia under submergence conditions and it promotes coleoptile elongation [8]. Auxin plays a major role in supporting coleoptile elongation by regulating early auxin-responsive genes as well as gravitropic growth [20,21,22,23,24]. ABA suppresses seed germination, and GA acts as the antagonist of ABA [25,26]. During seed germination, a decrease in ABA levels and a rise in GA biosynthesis are well documented in rice and many other plant species [18,27,28]. GA promotes the degradation of starch that provides substrates for rice germination and coleoptile elongation under submergence. JA, SA, and BR influence coleoptile growth by mediating the stress response associated with submergence and oxidative stress; however, much of their influence depends on ethylene signaling. JA inhibits coleoptile elongation; however, ethylene inhibits JA biosynthesis and, thus, indirectly supports coleoptile elongation. BR promotes coleoptile elongation [18,27,28].

Phytohormone signaling drives the transcriptional activation or differential expression of a master set of transcription factors (TFs), which play a crucial role in converting a stress signal to stress-responsive gene expression. TFs interact with *cis*-acting elements present in the promoter regions of their target genes to activate/repress/modulate their expression. Many TFs can also regulate self-transcription. Often, genes that participate in related processes are regulated by common TFs to activate the more extensive network of genes to bring change coherently. Previous studies have identified the major transcription factors Sub1A, SNORKEL 1, and SNORKEL 2 which confer submergence tolerance in rice at the vegetative stage in flood-tolerant rice varieties [11,29,30]. In addition, several candidate genes which show differential expression in response to submergence, hypoxia stress, and anaerobic germination have been identified in rice [1,12,24,31,32]. In general, the mechanisms employed for plant survival during the early stage of seed germination seem different when compared to mechanisms conferring submergence tolerance at the later vegetative stage. For instance, rice landrace Flood Resistant 13A (FR13A) that contains the *Sub1A-1* functional allele shows high tolerance to flooding at the vegetative stage. However, it shows low tolerance to submergence and hypoxia during seed germination and the coleoptile elongation stage. Likewise, several rice varieties lacking *Sub1A* are submergence intolerant at the vegetative stage but display a higher rate of anaerobic seed germination and coleoptile elongation under submergence (for a recent review, see [8]).

In recent years, genomic and transcriptomic studies in submergence-tolerant rice varieties [11,19,24,29] have been conducted to identify candidate genes involved in conferring submergence tolerance at various stages of plant development. However, these data have not been fully exploited to gain new information about gene functions, gene–gene interactions, and how genotypic differences relate to plant pathways and processes. We have previously shown that re-analyzing transcriptomic data can help identify genes associated with important abiotic and biotic responses and improve functional annotation of gene family members [1,33]. In this study, by re-analyzing transcriptome data generated by Hsu and Tung, 2017 [12], we identified a co-expression network of 57 TFs involved in the submergence response during seed germination and coleoptile elongation in tolerant rice genotypes. Furthermore, we conducted targeted promoter analysis to score potential TF-target relationships within this group of 57 TFs and reviewed the published scientific literature for gene–gene/gene–protein/protein–protein interaction. Finally, using the Plant Reactome data model, we manually curated a TF network based on the co-expression profile; the presence of a TF binding site within the promoter region of target genes; and published experimental evidence. We note here that the original generator of this dataset only focused on the gene ontology (GO)-based functional annotation of differentially expressed genes (DEGs) and did not conduct gene co-expression or gene network analysis or promoter analysis.

The Plant Reactome (https://plantreactome.gramene.org) is an online, free-of-cost plant pathway knowledgebase. It hosts and supports in silico modeling of plant pathways (i.e., metabolic, transport, gene-regulatory, hormone signaling) and processes (i.e., plant growth, organ development, cell cycle, and response to biotic and abiotic stresses). We have previously described the standard biocuration of plant pathways based on published empirical evidence using the Reactome Data Model [34,35,36,37]. Here we demonstrate how publicly available transcriptome and promoter sequence data can be re-used/re-analyzed for elucidating gene–gene interaction networks by extending the literature review-based manual biocuration approach. We expect this study promotes the findable, accessible, interoperable, and re-usable (FAIR) policy and practice of OMICs data to synthesize new knowledge related to complex biological processes.

## 2. Results

### 2.1. Co-Expression Analysis of Transcription Factor Genes during Rice Seed Germination and Coleoptile Elongation under Submergence

**Figure 1:**
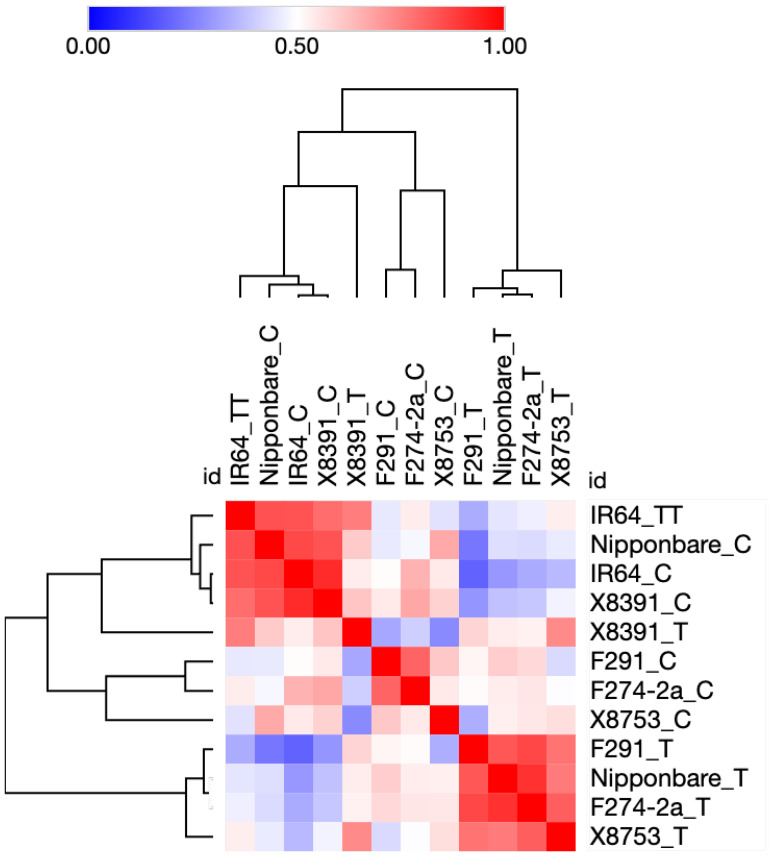
An average sample matrix displaying hierarchical clustering of rice genotypes based on the expression of 183 transcription factors during seed germination and coleoptile growth under submergence stress. Similarity scores are represented by colors, with red indicating high similarity and blue indicating low similarity. The log10 values of the data from Hsu and Tung, 2017 [12] were used for data visualization using the MORPHEUS tool (https://software.broadinstitute.org/morpheus). ‘C’ denotes controls. ‘T’ denotes samples of tolerant genotypes treated with submergence stress, and ‘TT’ is a treatment sample of the IR64 susceptible genotype. Clustering was performed among both selected genes and across different samples.

First, we updated the functional annotations of all 2026 DEGs identified by Hsu and Tung, 2017 [12] based on the current information available at the Rice Annotation Project database (RAP) (https://rapdb.dna.affrc.go.jp) [38], and Gramene (https://www.gramene.org) [39] (both databases accessed on 10 December 2022). Next, we extracted transcriptome data for 183 genes encoding for TFs. The expression profile of 183 TF genes suggested a clear difference between the submergence-susceptible IR64 rice genotype and the other five submergence-tolerant genotypes Nipponbare, F291, F274-2a, X8391, and X8753. As shown in Figure 1, the average gene expression profile of IR64 does not change significantly when subjected to submergence. IR64 samples subjected to submergence stress resemble Nipponbare controls (not subjected to submergence stress). In contrast, Nipponbare, F291, F274-2a, X8391, and X8753 show significant change in their overall gene expression under submergence compared with their respective controls. In addition, Nipponbare, F291, F274-2a, and X8753 share the majority of upregulated and downregulated genes in response to submergence. Notably, the expression profile of X8391 genes significantly differs from those of Nipponbare, F291, and F274-2a. Overall, X8391 is more similar to X8753 (see Figure 1).

Additionally, the comparison of expression profiles of 183 TFs revealed strong positive and negative correlations between various genes (see Supplementary Figures S1 and S2). We identified 57/183 genes that show a strong correlation in their expression during seed germination and coleoptile elongation under submergence (see Supplementary Figure S1). In this list, 41/57 genes were not identified as TFs (described as genes of unknown function) in the original study [12] or in another study that re-used this transcriptome data [40]. Thus, updating the gene functional annotations helped us to identify an important set of transcription factors that were ignored in two previous publications (see Table 1).

**Figure 2:**
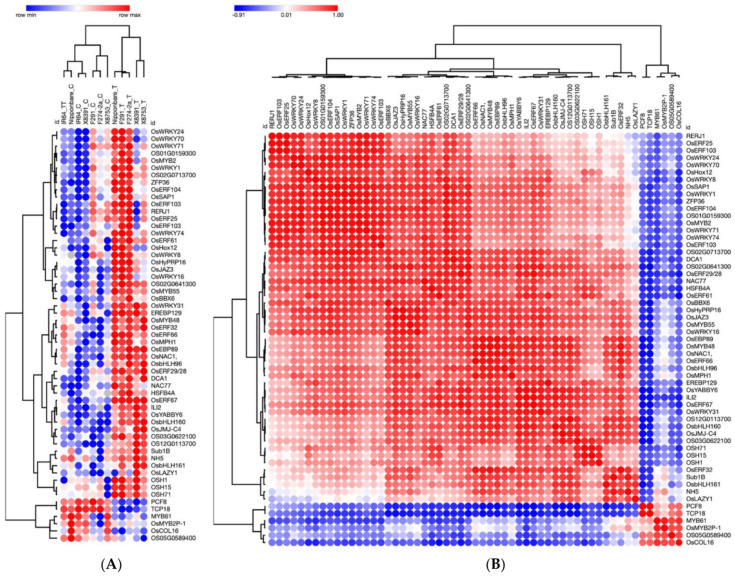
(**A**) A heatmap showing hierarchical clustering of 57 transcription factor genes showing strong coexpression correlations during seed germination and coleoptile grown under submergence across six rice genotypes. ‘C’ denotes controls, ‘T’ denotes samples of tolerant genotypes treated with submergence stress, and ‘TT’ is IR64 (susceptible genotype) treated with submergence stress. (**B**) Sample matrix showing 57 transcription factor genes clustering based on their gene expression profile. For both (**A**) and (**B**), log10 values of the data from Hsu and Tung, 2017 [12] were used for data visualization using the MORPHEUS tool.

To gain a more profound understanding of the co-expression profiles of 57 TFs, we visualized their expression profiles. As shown in Figure 2A, no significant difference in gene expression was observed in the susceptible genotype IR64 control (not subjected to submergence stress) and submergence-treated IR64 samples. In fact, IR64 submergence-treated samples resemble the Nipponbare control samples. However, 38/57 genes are upregulated in all submergence-tolerant rice genotypes, including Nipponbare, F291, F274-2a, X8391, and X8753 (see Figure 2A). Overall, the gene expression profile of Nipponbare, F291, and F2742a are more similar: these genotypes share 50 upregulated and four downregulated genes. The expression profiles of rice genotypes X8753 and X8391 are highly similar as they share 42 upregulated genes (see Figure 2A). Although most genes in this group show upregulation in response to submergence in tolerant genotypes, six genes, including *OS05G0589400*, *PCF8*, *TCP18*, *MYB61*, *OsMYB2P-1*, and *OsCOL16*, are downregulated in all tolerant genotypes (see Figure 2A). The hierarchical clustering of 57 genes, based on their expression across all samples, suggests a strong positive correlation between the upregulated genes and a strong negative correlation between the upregulated and downregulated genes (see Figure 2B).

Additionally, the comparison of control and submergence-treated samples from each genotype revealed several unique combinations of up- and down-regulated genes (as shown in Figure 2B and Supplementary Figure S2). Thus, it is likely that the combination of shared and unique differentially expressed genes in each rice genotype plays a role in its overall transcriptomic reconfiguration under submergence stress, ultimately leading to differential coleoptile elongation. This variation in coleoptile elongation and erectness may have implications for the survival of seedlings at early stages. As previously reported by Hsu and Tung, 2017 [12], the coleoptile elongation of IR64 is severely reduced under submergence. In contrast, in Nipponbare, F291, and F274-2a, coleoptile elongation remains more or less unaffected and is slightly enhanced in the genotypes X8391 and X8753 [12]. Nipponbare, Nipponbare/IR64-derived F291, and F274-2a are moderately tolerant to submergence stress [12]. The rice landraces X8391 and X8753 are extremely tolerant to submergence stress, and their coleoptile length was twice the length seen in genotype IR64 seedlings.

### 2.2. Key Transcription Factors Integrate Multiple Signaling Pathways to Regulate Rice Seed Germination and Coleoptile Elongation under Submergence

Gene co-expression information is often used to construct gene networks to understand the transcriptional regulation at a genome-wide level and to link genes that may have common regulators and/or play a role in closely related processes [41,42,43]. We carefully note here that the evidence from the co-expression of genes in the same tissues and organs and in response to a given stress condition only suggests possible connections among these genes. Thus, to establish the direct or indirect ‘Transcription Factor–Target’ relationship within this group of 57 TFs, we investigated transcription factor binding sites present in the promoter region of each of these genes (see Supplementary Table S1). In addition, published empirical evidence on the promoter–TF bindings, gene–protein/protein–protein interactions, and mutants were gathered to support gene–gene connections and their associations to pathways associated with rice plant development as well as a stress response (see Table 1 for summaries of all TFs). Overall, we found that 26/57 TFs have one or more binding sites in the promoter region of many genes. The remaining 31 TFs have no binding site present in the promoter region of any genes included in this group (see Supplementary Table S1).

**Table 1:** A list of 57 transcription factors (TFs) that show a strong correlation in their expression profile in response to submergence during rice seed germination and coleoptile elongation in tolerant rice genotypes. The 41 genes marked with ‘*’ were not identified as TFs in the previous studies [12,40]. “^$^” denotes 31 TFs that do not have binding sites in the promoter regions of the 57 genes included in this study. TFs that show down-regulation in their transcription under submergence in tolerant rice genotypes during seed germination and coleoptile elongation are underlined.

RAP Gene ID and Gene Symbols	UniProt Protein ID	Gene Summary
**TFs involved in shoot apical meristem, and early embryonic development**		
Os03g0727000 * *OSH1*, *Oskn1*,	P46609	*Oryza sativa* Homeobox protein 1 (OSH1) plays a key role in the development and maintenance of the shoot apical meristem (SAM) [44,45,46] as well as in morphogenetic processes throughout plant development [47,48]. It is involved in the regionalization of cell identity and expresses before the development of SAM in the mature embryo. During postembryonic organ development, SAM differentiates into all aerial organs, such as leaves, stems, and flowers. The expression of the *OSH1* gene is autoregulated to maintain SAM [49]. It is upregulated in response to submergence during seed germination and coleoptile elongation (this study).
Os07g0129700 * *OSH15*, Oskn3, *OsKNOX15*, *HOS3*,	O80416	*Oryza sativa* Homeobox protein 15 (OSH15) is involved in shoot formation, internode development, repression of lignin biosynthesis, and control of seed shattering [50]. Loss-of-function mutants of *OSH15* have a *d6*-type dwarf phenotype [51]. It was upregulated in response to submergence during seed germination and coleoptile elongation (this study).
Os05g0129700 *OSH71*, *Oskn2*, *HOS9*	Q7GDL5	*Oryza sativa* Homeobox protein 71 (OSH71) is involved in shoot formation during embryogenesis [47,52]. Its ectopic expression induced defects in panicle branching, internode elongation, and leaf patterning [53]. It was upregulated in response to submergence during seed germination and coleoptile elongation [12].
**TFs involved in plant growth and development, shoot architecture, and gravitropism**		
Os12g0138500 *^$^ *NH5*, *OsBOP3*	Q2QXZ2	NPR1 Homolog 5 (NH5), also known as Blade-On-Petiole3 (OsBOP3), controls the shape of the first leaf and ligule formation in rice [54]. *OsBOP1, OsBOP2,* and *OsBOP3* functions are redundant in rice: these TFs promote leaf sheath differentiation while repressing blade differentiation. *OsBOP1* and *OsBOP2/3* expression was highest in the first leaf primordia, in which blade differentiation is strongly inhibited, and show decrease in the second leaf primordium [54].
Os10g0456800 **^$^** *DCA1*	Q337P2	DST Co-Activator 1 (DCA1) is upregulated in response to submergence during seed germination and coleoptile growth [12]. Previous studies have shown that DCA1 and Drought and Salt Tolerance (DST) form a hetero-tetrameric transcriptional complex that promotes stomatal opening in the guard cells and promotes transpiration, thus, negatively regulating drought and salt tolerance [55].
Os12g0621100 *^$^ *OsYABBY6*	Q2QM17	OsYABBY6 is a member of the plant-specific transcription factor family. It regulates the initiation and development of the leaf blade (the development of vascular bundles, mestome sheath, and sclerenchyma) by suppressing the expression of meristem-specific genes in leaf primordia [56,57]. It is upregulated in response to submergence during seed germination and coleoptile elongation (this study).
Os06g0670300 *^$^ *OsMPH1*, *OsMYB45*	A0A0P0X0C0	The Rice MYB-like gene of Plant Height 1(OsMPH1) acts as a positive regulator of plant height by elongating internodal cell length and cell wall synthesis. It shows extremely high expression in the leaves (sheaths and especially in the pulvinus), and low expression in the internodes and spikes [58]. It also promotes large panicle size and grain yield [58]; and plays a key role in the tolerance to Cd stress in rice [59]. It is upregulated in response to submergence during seed germination and coleoptile elongation (this study).
Os03g0198600 **^$^** *OsHox12*, *HOX12*	Q10QF2	Homeobox gene 12 (HOX12) is a negative regulator of internode growth. HOX12 positively regulates transcription of *Elongated Uppermost Internode1* (*EUI1*) by directly binding to the *EUI1* promoter [60]. EUI1 is a GA-deactivating enzyme. Inactivation of *HOX12* or *EUI1* results in higher GA4 levels in the uppermost internode, promoting cell division and elongation. The *HOX12* RNAi plants and *eui1* knockdown mutants show similar phenotype: enhancement in panicle exsertion due to elongation of the uppermost internode.
Os06g0264200 *^$^ OsCOL16, OsBBX17	Q0DD26	Constans-like 16 (OsCOL16) shows higher expression in vegetative tissues than in reproductive tissues. In the vegetative stage, it pro-motes plant height and delays flowering. During the reproductive stage, it promotes grain yield [61]. It is downregulated in response to submergence during seed germination and coleoptile elongation in sub-mergence-tolerant rice genotypes (this study).
Os02g0713700 *^$^	Q6ZFU2	Os02g0713700 is a DUF296 domain-containing TF [61]. It shows differential expression in response to imbalanced Carbon: Nitrogen availabilities [62]. It is upregulated in response to submergence during seed germination and coleoptile growth (this study).
Os01g0285300 *^$^ MYB61, OsMYB61	Q9AQV2	OsMYB61 is involved in the regulation of cellulose synthesis, nitrogen assimilation, carbon fixation, and growth. It works in the same pathway as Growth-Regulating Factor 4 (GRF4) [63]. It connects Carbon and Nitrogen metabolism in rice [63]. During seed germination and coleoptile growth, OSMYB61 is downregulated in response to submergence in tolerant rice genotypes (this study).
Os05g0140100 ^$^ OsMYB2P-1	Q688D6	MYB2 phosphate-responsive gene 1 (OsMYB2P-1) regulates phosphate-starvation response and root architecture in rice [64]. It is downregulated in response to submergence during seed germination and coleoptile growth in the tolerant rice genotypes (this study).
Os11g0490600 *^$^ *OsLAZY1*	Q2R435	OsLAZY1 regulates shoot gravitropism and tiller angle through negative regulation of basipetal polar auxin transport (PAT) and positive regulation of lateral auxin transport (LAT) that results in enhancing vertical shoot growth [65,66,67,68,69,70]. *OsLAZY1* is mainly expressed in gravity-sensitive shoot tissues such as coleoptiles, leaf sheath pulvini, and lamina joints [67] and less expressed in roots. It is expressed specifically in the cells at the inner side of the vascular bundles of young leaf sheaths and peripheral cylinders of vascular bundles in the unelongated stems [66]. It is upregulated in response to submergence during seed germination and coleoptile growth [12].
Os11g0603000 *^$^ *ILI2*	Q2R1J3	Increased Leaf Inclination 2 (ILI2), a bHLH transcription factor, is mainly expressed in the lamina joint during leaf development and negatively regulates leaf angle. The *lc2* mutants have enlarged leaf angles. It is induced by ABA, GA, auxin, and BR [71]. Furthermore, it is upregulated in response to submergence during seed germination and coleoptile growth (this study).
**TFs involved in response to submergence and other abiotic and biotic stress conditions**		
Os09g0287000 * Sub1B, *EREBP166*, *OsERF#063, OsERF63*	Q6EN65	Submergence-1B (Sub1B) is a member of the ERF family. It is induced by drought, salinity, and submergence in rice seedlings [72]. It is upregulated in response to submergence during seed germination and coleoptile growth (this study).
Os03g0341000 * *OsERF66*, *EREBP30*	Q10LN8	*OsERF66* is a direct transcriptional target of Sub1A in some submergence-tolerant indica rice genotypes. Sub1A, OsERF66, and OsERF67 form a regulatory cascade, and both OsERF66 and OsERF67 are substrates of the N-end rule pathway and promote submergence tolerance of rice seedlings [31,73]. OsERF66 is upregulated in response to submergence during seed germination and coleoptile growth (this study).
Os07g0674800 * *OsERF67*	Q69J87	*OsERF67* is a direct target of Sub1A. Together with OsERF66, it functions downstream of Sub1A to form a regulatory cascade in response to submergence stress. It is a substrate of the N-end rule pathway and enhances transcriptional response to increasing submergence survival in rice [31,73]. In addition, it is upregulated in response to submergence during seed germination and coleoptile growth (this study).
Os05g0361700 * *OsERF61*, *EREBP93*	Q6L4M2	OsERF61 regulates rice growth and metabolism in response to abiotic stresses [74]. It is involved in the OsDRAP1-mediated regulation of transcription in response to salt stress [72]. It is upregulated in response to submergence during seed germination and coleoptile growth (this study).
Os02g0656600 *OsERF32*, *OsERF032*, *EREBP21*	Q6H6G7	OsERF32 is induced by drought, high salt, high temperature, and in response to submergence during seed germination and coleoptile growth [12,75,76,77].
Os02g0677300 *OsDREB1G*, *OsERF25*, *EREBP138*	Q6EP77	Dehydration-Responsive Element-Binding 1G (OsDREB1G) is involved in both abiotic and biotic stress tolerance in rice [12,76,78,79,80,81]. It expresses in leaf sheath, blade, internode, and roots [78]. It binds to the promoters containing DRE elements to activate stress-responsive genes [76]. In addition, its expression is upregulated in response to submergence during seed germination and coleoptile growth [12].
Os08g0545500 * OsERF28, *OsERF29*, *OsDERF4*, *EREBP161*	Q0J3Y6	OsERF29 is in a co-expression network of TFs which enhance drought, cold, and salt tolerance in rice [76,78,81]. Its expression reaches the highest level at 18 h after seed imbibition, and then decreases gradually [82]. Its expression is upregulated in response to submergence during seed germination and coleoptile growth (this study).
Os02g0764700 * *OsERF103*, *OsDERF5*, *EREBP130*	Q6Z7P9	OsERF103 negatively regulates ethylene biosynthesis and drought stress tolerance in rice. Its expression is also upregulated at the reproductive stage in rice [83]; and in response to submergence during seed germination and coleoptile growth (this study).
Os08g0474000 * *EREBP152*, *OsERF104*, *OsDERF3*,	Q0J525	OsERF104 is known to be regulated by OsNAC45 in response to salt [84]. It is upregulated in response to submergence during seed germination and coleoptile growth (this study).
Os02g0676800 *^$^ *OsERF20* *OsDREB1E*	Q6EP81	OsERF20 positively regulates chilling tolerance in rice seedlings by controlling ROS scavenging and reducing cell death [81]. It is also upregulated in response to submergence during seed germination and coleoptile growth (this study).
Os01g0141000 * EREBP129, *OsRAV2*, OsRAV9, OsTEM1	Q9AWS0	Ethylene-Responsive Element Binding Protein 129 (EREBP129) is regulated in response to abiotic and biotic stresses [85,86,87]. It acts as a repressor of photoperiodic flowering in rice [85]. It is upregulated in response to submergence during rice seed germination and elongation of the coleoptile; and it is likely to regulate the expression of *OSH1* and many other TF genes (this study).
Os03g0437200 *ZFP36*, *OsBSRD1*, *OsDLN91*	Q75KE5	Zinc Finger Protein 36 (ZFP36) regulates ABA-mediated abiotic stress response through reactive oxygen species signaling and promotes oxidative stress tolerance [88]. It is upregulated in response to submergence during seed germination and coleoptile growth [12].
Os02g0579000 * *OsNAC1*, *ONAC27*, *OMTN1*, *OsDLN60*	Q0E046	Rice NAC domain-containing protein 1 (OsNAC1) shows higher expression in the stamen, leave blade, embryo, root, and panicle. Its expression is downregulated in response to drought; and upregulated in response to cold stress and ABA treatment [89] and in response to submergence during seed germination and coleoptile growth (this study).
Os12g0123800 * *NAC77*, *NAC077*, *ONAC300*, *OsNAC132*, *OsDLN250*	Q5CD17	Rice NAC domain-containing transcription factor 77 (NAC77) expresses at early developmental stages in the shoot, root, flower, and the mature phloem of vascular tissues [90]. It is upregulated in response to both abiotic (salt, drought, and cold) and biotic stresses in rice [91,92]. In addition, it is upregulated in response to submergence during seed germination (this study).
Os03g0182800 *^$^ *OsEBP89*	Q7XBH8	Ethylene-responsive element binding protein 89 (OsEBP89) is a negative regulator of ABA-dependent stress responses [93]. Its expression is strongly inhibited by drought stress and stimulated by submergence stress in the root and meristem [93]. We find that it is upregulated during seed germination and coleoptile elongation under submergence rice (this study). *OsEBP89* knockout mutant showed improved seed germination rate under submerged conditions and, thus, enhances direct seeding on wetlands and confers drought tolerance in rice [93]. OsEBP89 is phosphorylated by sucrose non-fermenting-1-related protein kinase-1 (OsSnRK1α) [93].
Os01g0246700 * *OsWRKY1*	Q0JP37	OsWRKY1 is upregulated in response to submergence during seed germination and coleoptile growth (this study).
Os05g0583000 *OsWRKY8*	Q75HY3	OsWRKY8 is induced by PEG, NaCl, ABA, and naphthalene acetic acid in rice. It is upregulated in response to submergence during seed germination and coleoptile growth [12].
Os01g0665500 * *OsWRKY16*	Q0JKL7	OsWRKY16 is upregulated in response to submergence during seed germination and coleoptile growth (this study).
Os01g0826400 * *OsWRKY24*	Q6IEQ7	OsWRKY24 acts as both a transcriptional repressor and an activator. It is expressed chiefly in aleurone cells and embryos within the seeds [94] and shows low expression in leaves, roots, and panicles [95]. Its expression is induced by ABA and JA but repressed by GA [94,96]. It is upregulated in response to submergence during seed germination and coleoptile growth (this study).
Os03g0321700 * *OsWRKY31*, *OsWRKY55*	Q10M65	OsWRKY31 is a component of the auxin signaling pathways and is also involved in the defense response in rice [97]. It is upregulated in response to submergence during seed germination and coleoptile growth (this study).
Os05g0474800: *OsWRKY70*	Q65WW1	OsWRKY70, together with OsWRKY24, OsWRKY53 acts as a negative regulator of both GA and ABA signaling [96]. OsWRKY70 positively regulates early defense signaling by inducing the synthesis of the volatile indole [98]. It is also upregulated in response to submergence during seed germination and coleoptile growth [12].
Os02g0181300 * *OsWRKY71*	Q6QHD1	OsWRKY71 expression is induced by SA, JA, 1-aminocyclo-propane-1-carboxylic acid, wounding, and pathogen infection. It is likely to function as a transcriptional regulator of OsNPR1 and OsPR1b in rice defense signaling pathways [99].
Os09g0334500 * *OsWRKY74*	Q6ERI5	OsWRKY74 expresses in roots and leaves. It is involved in the tolerance to phosphate starvation and shows differential expression in response to iron and nitrogen deficiencies, and cold stress [100]. It is upregulated in response to submergence during rice seed germination and coleoptile growth (this study).
Os03g0315400 *^$^ *OsMYB2*	Q10MB4	OsMYB2 is a positive regulator of salt, cold, and dehydration tolerance in rice [101]. Overexpression of *OsMYB2* leads to the accumulation of soluble sugars and proline, and a decline in the levels of H_2_O_2_ and malondialdehyde. OsMYB2 overexpressing plants also showed upregulation of stress-related genes, including *OsLEA3*, *OsRab16A*, and *OsDREB2A* [101]. In addition, it is upregulated in response to submergence during seed germination and coleoptile growth in rice (this study).
Os05g0553400 **^$^** *OsMYB55* *OsPL9*	Q6I634	OsMYB55 confers heat tolerance in rice by increasing the biosynthesis of glutamic acid, proline, arginine, and GABA [102]. Under heat stress, rice coleoptiles that overexpressed *OsMYB55* became longer [102] and showed higher plant growth and grain yields than the wild type. It also enhances anthocyanin biosynthesis in rice [103]. In addition, it is upregulated in response to submergence during seed germination and coleoptile growth [12].
Os01g0975300 *^$^ *OsMYB48*, *OsMYB48-1*, *OsMYB48-2*	Q0JFK3	*OsMYB48* is strongly induced by polyethylene glycol, ABA, H_2_O_2_ and dehydration, and slightly induced by high salinity and cold. It is a positive regulator of drought and salinity tolerance [104]. It is upregulated in response to submergence during seed germination and coleoptile growth in rice [12].
Os02g0641300 *^$^ *OsMYB21*	Q0DZ73	*Os02g0641300/OsMYB21* is upregulated in response to chilling stress [105]. Also, it is induced in response to submergence during seed germination and coleoptile growth in rice (this study).
Os05g0589400 *^$^	Q6I5E6	Os05g0589400 encodes a R2R3MYB transcription factor. It is downregulated in response to submergence during seed germination and coleoptile growth in submergence-tolerant rice genotypes (this study).
Os08g0471000 **^$^** *HSFB4A*, *OsHsf-20*	Q6Z9R8	Heat stress transcription factor B-4a (HSFB4A) could act as a seed-specific transcriptional repressor co-activator in rice [106,107,108]. It is likely to form homotrimer. It is upregulated in response to submergence during seed germination and coleoptile growth [12].
Os03g0188400 **^$^** *OsbHLH96*	Q8H7N8	Rice basic helix-loop-helix 96 (OsbHLH96) forms the OsbHLH96-OsHLH61 complex that regulates PR genes in response to brown planthopper attack and is likely to be involved in mediating crosstalk between SA and JA signaling. OsbHLH96 could also interact with OsJAZ3 [109]. In addition, OsbHLH96 is upregulated in response to submergence during seed germination and coleoptile growth [12].
Os11g0111800 *^$^ *OsbHLH160*	A3C7W7	OsbHLH160 expresses in rice roots [110]. It is upregulated in response to submergence during seed germination and coleoptile growth (this study).
Os12g0111400 *^$^ *OsbHLH161*	Q2QYP2	*OsbHLH161* [111] is upregulated in response to submergence during seed germination and coleoptile growth (this study).
Os04g0301500 *^$^ *OsbHLH6*, *RERJ1*	Q0JEB7	OsbHLH6 is induced by JA, wounding, drought, and Pi deficiency [112,113]. It acts as a positive regulator for Pi signaling and homeostasis. It is also upregulated in response to submergence during seed germination and coleoptile growth (this study).
Os09g0486500 **^$^** *OsSAP1*, *ISAP1*	A3C039	Rice Stress Associated 1 (*OsSAP1*) expression is induced by cold [114] and submergence stress during seed germination and coleoptile growth [12]. It regulates biotic and abiotic stress responses [115].
Os04g0612500 *^$^ *OsHyPRP16*	B9FCG2	Hybrid Proline-Rich Protein 16 (*OsHyPRP16*) is upregulated in response to *M. oryzae* infection [116]. It is also upregulated in response to submergence stress during seed germination and coleoptile growth (this study).
Os01g0159300 *^$^	Q0JQI1	Os01g0159300 is upregulated in response to submergence during seed germination and coleoptile growth (this study).
Os02g0646200 **^$^** *OsBBX6*	Q6H630	The Rice B-box domain containing 6 (OsBBX6) is a member of the Zinc Finger TF family [117]. It is upregulated in response to submergence during seed germination and coleoptile elongation [12].
Os12g0113700 **^$^**	A0A0P0Y689	Os12g0113700 is a Zinc finger C3HC4 type TF. It is also upregulated in response to submergence during seed germination and coleoptile growth [12].
Os02g0828900 *^$^ *OsJMJ-C4*	Q6K7P0	OsJMJ-C4 (Jumonji C domain-containing protein 4) is likely to function as histone demethylases or a TF involved in the epigenetic regulation of plant development [118,119]. It is upregulated in response to submergence during seed germination and coleoptile growth (this study).
Os08g0428400 **^$^** *OsJAZ3*, *TIFY6a*	Q6ZJU3	OsJAZ3 repressor is induced by ABA, drought, and salt stresses. It is targeted for degradation by the SCF (COI1) E3 ubiquitin ligase–proteasome pathway during JA signaling [120,121]. It is upregulated in response to submergence during seed germination and coleoptile growth [12].
Os03g0622100 *^$^	Q10GM4	Os03g0622100, a B3 family TF, is upregulated in response to submergence during rice seed germination and coleoptile growth (this study).
Os12g0616400 * PCF8	Q2QM59	Proliferating Cell Factor 8 (PCF8) acts as a negative regulator of cold tolerance in rice, and its expression is downregulated by miR319 [122]. Furthermore, it is downregulated in response to submergence during seed germination and coleoptile growth in the tolerant genotypes (this study).
Os05g0513100 * OsTCP18, TCP18	Q5TKH1	Teosinte branched 1, Cycloidea and Proliferating 18 (TCP 18) could act as transcriptional activators or repressors [123]. However, it is downregulated in submergence-tolerant genotypes of rice in response to submergence during seed germination and coleoptile growth (this study).

#### 2.2.1. Transcription Factors involved in Shoot Apical Meristem Maintenance, and Coleoptile Elongation

**Figure 3:**
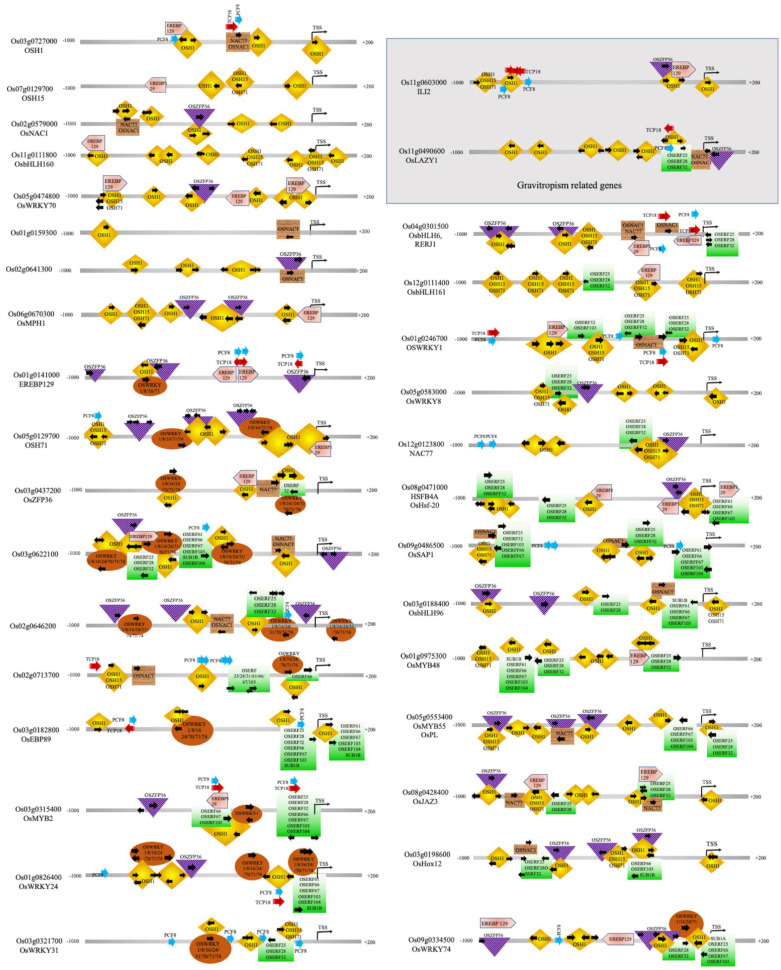
Promoter analysis of *OSH1* gene and genes regulated by OSH1 TF.

**Figure 4:**
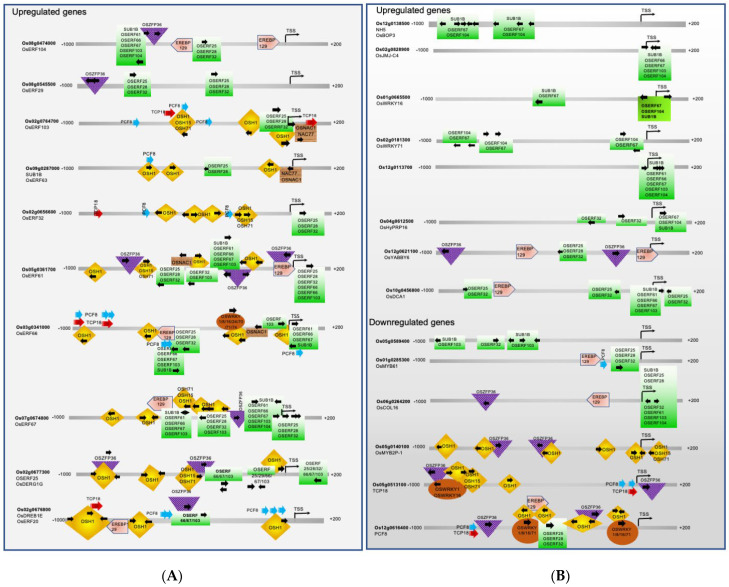
Promoter analysis of ERF-regulated genes that show differential expression during rice seed germination and coleoptile elongation. (**A**) Promoter analysis of ERFs coding genes. (**B**) Promoter analysis of ERFs-regulated genes that show upregulation, and promoter analysis of downregulated genes including four ERF-targets and two genes that are not direct targets of ERFs. The downregulated genes are shaded with a darker grey color.

As shown in Figure 3 and Figure 4, OSH1 is likely to play a dominant role in regulating seed germination and coleoptile development by regulating the expression of the majority of transcription factors. *OSH1*, a plant-specific homeobox gene, is involved in the regionalization of cell identity and the development of shoot apical meristem (SAM) in the mature embryo. In general, SAM differentiates into organs such as leaves and stems and transitions into reproductive shoot apical meristem (RSAM), giving rise to rice inflorescence and florets [44,45,46,47,48]. *OSH1* expression is high in the SAM, epiblast, radicle, and intervening tissues but gradually declines. *OSH1* has been shown to inhibit cell differentiation by activating BR catabolism genes [124,125,126]. The decline in OSH1 levels allows organogenesis to proceed; thus, *OSH1* acts as a master “ON and OFF” switch to control seed germination, coleoptile elongation, and setting the trajectory for subsequent organ differentiation. The promoter of the *OSH1* gene has four self-binding sites (see Figure 3), consistent with the previous finding that the expression of *OSH1* is autoregulated to maintain SAM [49]. Moreover, *OSH1* overexpression in transgenic rice plants caused altered leaf morphology, while its disruption led to early termination of shoot meristem, reduced leaf formation, and reduced seed setting [44,47,127].

OSH15 and OSH71 are two additional homeobox TFs that are upregulated during seed germination and are involved in shoot formation, internode development, and plant height [47,52]. Loss-of-function mutants of the *OSH15* gene have a d6-type dwarf phenotype in rice [51]. Double mutants of *OSH1* and *OSH15* fail to establish the SAM during embryogenesis [49]. As shown in Figure 3, the *OSH15* promoter has binding sites for OSH1, OSH71, and itself. OSH15 targets BR homeostasis and signaling genes, including *OsBRI1*, *BR SIGNALING KINASE1-2/2*, *GSK1/3*, *OsBZR2/3*, *D11,* and *OsCYTOCHROME P450 734A5/6* [128]. Previous studies have shown that *OSH71* ectopic expression induced defects in panicle branching, internode elongation, and leaf patterning [53]. As shown in Figure 3, the *OSH71* promoter has binding sites for OSH1, OSH15, and OSH71 as well as for ZFP36 and WRKYs TFs. Thus, OSH71 is likely to control organogenesis and likely to mediate abiotic submergence and hypoxia stress. The *OSH1, OSH15,* and *OSH71* have a common regulator EREB129 (see Figure 3). EREB129 is known to repress photoperiodic flowering [85] and mediate the stress responses in rice [86,87]. *EREB129* promoter also has a binding site for OSH1 and two binding sites for self-regulation. OsNAC1 and NAC71 are also regulators of *OSH1*. *OsNAC1* is also a target of OSH1 (but not a target of OSH15 or OSH71).

A previous study has shown that *OsNAC1* (referred to as *OMTN1*) shows higher expression in the stamen, leaf lamina, embryo, root, and panicle. Its expression is downregulated in response to drought and upregulated in response to cold stress and ABA treatment [89]. Rice NAC77 is known to express in the SAM [90] and is upregulated in response to salt, drought, and cold [91,92]. As shown in Figure 3, the rice *NAC77* promoter has binding sites for OSH1, OSH15, and OSH71 and many ERFs. OsZFP36 is a known regulator of ABA-mediated abiotic stress response through ROS signaling [88]. OsZFP36 has binding sites within the promoters of *OSH71*, *OsNAC1*, *NAC77*, *EREB129*, etc. Interestingly, *OsZFP36* genes is also regulated by OSH1, NAC77, and EREB129. Here we note that *OsZFP36* is upregulated and likely to regulate the expression of *OSH1* and other key TFs during seed germination and coleoptile growth under submergence. Previously, OsZFP36 was suggested to regulate seed germination by enhancing the expression of NADPH oxidase genes, ascorbate peroxidases I, and several kinases associated with the ROS signaling cascade, thus contributing to oxidative stress tolerance [129]. Thus, we find that OSH1, OSH15, OSH71, OsNAC1, NAC77, EREB129, and OsZFP36 are important regulators of early seed germination and embryonic growth stages, which provide the foundation for continued SAM maintenance, organ differentiation, and integration of the submergence and hypoxia stress response.

#### 2.2.2. Transcription Factors Involved in the Shoot, Leaf and Flower Development, and Gravitropism

We note here that the 31 TFs have no binding sites in the promoter region of any gene in the group of 57 genes (the focus of this study). These rice TFs are likely to regulate processes involved in later stages, such as organogenesis, plant architecture, and gravitropism. For instance, in this group, three upregulated TFs, OsYABBY6, OsBOP3, and DCA1, are known to play roles in leaf development and function. OsYABBY6 is likely to regulate the initiation and development of the leaf blade (i.e., the development of the vascular bundles, mestome sheath, and sclerenchyma) by suppressing the expression of meristem-specific genes in leaf primordia [56,57]. A previous study has shown that *OsBOP1, OsBOP2,* and *OsBOP3* redundantly control the shape of the first leaf and ligule formation in rice [54]. *OsBOP1* and *OsBOP2/3* expression was highest in the first leaf primordia, in which blade differentiation is strongly inhibited, followed by a sudden decrease in the second leaf primordium. Likewise, low expression of OsBOPs was observed in the second to fourth leaves [54]. The triple mutant of *OsBOP1-3* genes were extremely short plants containing dark and curled leaves that lacked ligules and auricles. *OsBOP1-3* genes control the proximal–distal patterning of rice leaves by promoting sheath differentiation and repressing lamina differentiation [54]. Previous studies have shown that DCA1 and Drought and Salt Tolerance (DST; *Os03g0786400*) form a heterotetrametric transcriptional complex that promotes stomatal opening in the guard cells and promotes transpiration, thus, negatively regulating drought and salt tolerance [55]. In response to various abiotic stresses, including drought, heat, and salinity, in the guard cells, ABA stimulates the accumulation of ROS. The ROS signaling promotes stomatal closure by activating plasma membrane calcium channels. The OsDCA1 and DST complex upregulates the expression of peroxidase 24 precursor (Prx24) that acts as an H_2_O_2_ scavenger in guard cells resulting in lowered accumulation of ROS. The decrease in ROS interferes with stomatal closure [55]. Thus, upregulation of DCA1 promotes stomata opening in the developing coleoptile. The promoters of all three genes, *OsYABBY6*, *OsBOP3,* and *DCA1*, lack a binding site for OSH1, OSH15, and OSH71; however, all three contain binding sites for ERFs. Both *OsYABBY6* and *DCA1* promoters contain EREBP129 binding sites. In addition, the *OsYABBY6* promoter has two binding sites for OsZFP36.

Similarly, in this subgroup, two genes, OsMPH1 and HOX12, are known to regulate internode elongation. They both lack binding sites within the promoters of other genes included in the group of 57 TFs. OsMPH1 acts as a positive regulator of internode elongation by promoting cell length and cell wall synthesis [58]. It promotes panicle size and grain yield [58], and regulates tolerance to Cd stress in rice [59]. In contrast, HOX12 acts as a negative regulator of internode growth by activating transcription of a GA-deactivating enzyme *EUI1* [60]. EUI1 is considered to function mainly in the uppermost internode. Inactivation of *HOX12* or *EUI1* results in higher GA4 levels in the uppermost internode, which in turn promotes cell division and/or cell elongation. Endogenous and exogenous ABA and GA induce *EUI1* expression. EUI1 has two homologs, CYP714B1 and CYP714B2, in rice. Compared with *EUI1*, *CYP714B1* and *CYP714B2* are highly expressed in spikelets and the uppermost internodes of mature rice plants [60]. In addition to internode elongation, HOX12 regulates panicle exertion from the flag leaf sheath in rice [60]. It expresses in seedlings, roots, stems, leaf sheaths, and panicles [130], and its expression is induced by salt, drought, cold as well as by ABA, indole-3-acetic acid, JA, and GA3. The promoters of both *HOX12* and *OsMPH1* contain binding sites for OSH1, OSH15, OSH71, and OsZFP36. In addition, the *HOX12* promoter has binding sites for OsNAC1 and ERFs, whereas the *OsMPH1* promoter has binding sites for EREBP129.

Interestingly, *OsCOL16* expression declines during seed germination and coleoptile elongation under submergence in tolerant rice genotypes. Under normal growth conditions, *OsCOL16* expression is higher in vegetative tissues than in reproductive tissues, and it is suggested to promote plant height and delay flowering [131]. OsCOL16 represses flowering by upregulating the expression of floral repressor gene *Ghd7* [131]. In addition, *OsCOL16* transcription is upregulated by GA, SA and JA, and heavy metals including Fe, Ni, Cr, and Cd [132]. OsCOL16 lacks binding sites in the promoters of all 57 TFs included in this study.

Two MYB family TFs, OsMYB61 and OsMYB2P-1, associated with nutrient utilization are downregulated in tolerant rice genotypes during seed germination and coleoptile growth under submergence. OsMYB61 connects carbon and nitrogen metabolism in rice [63] by regulating cellulose synthesis, nitrogen assimilation, and carbon fixation. The robust transcription of *OsMYB61* in the indica allele increases biomass, nitrogen-use efficiency and grain yield under low nitrogen conditions compared with the japonica variety. The expression of *OsMYB61* is positively regulated by GRF4 [63] and GA [133]. In contrast, *OsMYB2P-1* expression is shown to be induced by Pi starvation, and its overexpression enhanced tolerance to Pi starvation and led to an increase in primary root length. In contrast, *OsMYB2P-1* silencing in RNAi transgenic rice lines resulted in more sensitivity to Pi deficiency [64]. It is likely that these two MYBs promote root function in nutrient uptake and utilization which occurs after coleoptile formation. Moreover, the erect and elongated coleoptile determines the plant’s survival under submergence; thus, it is likely that in tolerant rice genotypes, coleoptile growth is supported at the expense of root formation. The *OsMYB2P-1* promoter only has several binding sites for OSH1, OSH15, OSH71, and OsZFP36. Thus, it is less likely to be regulated directly in response to submergence stress. On the other hand, the *OsMYB61* promoter has binding sites only for EREBP129, ERFs, and PCF8, suggesting that it may be directly regulated in response to submergence stress. Both OsMYB61 and OsMYB2P-1 lack binding sites in the promoter regions of any gene included within the group of 57 TFs.

We also find two genes associated with gravitropism, *OsLAZY1* and*(ILI2,* that show increased expression during seed germination and coleoptile growth in submergence tolerant rice genotypes. OsLAZY1 controls shoot gravitropism and tiller angle through negative regulation of basipetal polar auxin transport (PAT) and positive regulation of lateral auxin transport (LAT), enhancing vertical shoot growth [65,66,67,68,69,70]. Previous studies have shown that *OsLAZY1* expresses in gravity-sensitive shoot tissues such as coleoptiles, leaf sheath pulvini, and lamina joints [67] and shows less expression in roots. It specifically expresses in the cells at the inner side of the vascular bundles of young leaf sheaths and the peripheral cylinders of vascular bundles in the unelongated stems [66]. ILI2 is a bHLH transcription factor known to be expressed in the lamina joint during leaf development and negatively regulates leaf angle. It is induced by ABA, GA, auxin, and BR [71]. Both *OsLAZY1* and *ILI2* are the target of OSH1. Thus, these two genes involved in gravity sensing are among the very first genes expressed during seed germination and coleoptile growth. In addition, the promoters of *OsLAZY1* and *ILI2* contain binding sites for OsZFP36, PCF8, and TCP18. The *OsLAZY1* promoter also contains binding sites for OsNAC1, NAC77, and ERFs. The *ILI2* promoter has additional binding sites for EREB129, OSH15, OSH71, and OsZFP36.

In conclusion, we find OsYABBY6, OsBOP3, DCA1, OsMPH1, HOX12, OsCOL16, OsMYB61, and OsMYB2P-1 are important TFs for the initiation of organogenesis, while OsLAZY1*,* and ILI2 are important TFs for sensing and regulating gravitropism during seed germination and coleoptile elongation under submergence in rice. Although the role of these genes has been suggested in previously published studies, our analysis suggests how common TFs regulate these genes.

#### 2.2.3. TFs Involved in Stress Response during Seed Germination under Submergence in Rice

As shown in Figure 2 and described in Table 1, we have found many different classes of stress response TFs (i.e., ERFs, WRKYs, MYBs, TCP, PCF, etc.) that show change in their expression during rice seed germination and coleoptile growth under submergence. Ethylene has been identified as a major signaling molecule that triggers the submergence response in plants. Previous studies in rice have shown the presence of higher levels of endogenous ethylene in the coleoptiles of flooding-tolerant rice genotypes compared with intolerant genotypes [14]. Furthermore, *Sub1A*, the major gene responsible for conferring flooding tolerance in some indica rice genotypes at the vegetative stage, is a member of the ERF family [29]. It has been shown that OsERF66 and OsERF67 are the direct transcriptional targets of Sub1A in submergence-tolerant indica rice genotypes. Sub1A, OsERF66, and OsERF67 form a regulatory cascade in response to submergence stress in rice. Both OsERF66 and OsERF67 are substrates of the N-end rule pathway and are involved explicitly in regulating the hypoxia stress response associated with submergence [31,73]. Sub1A has not been associated with submergence tolerance during seed germination and the coleoptile elongation stage. The transcriptome data re-used in this study do not include data for Sub1A. However, *OsERF66* and *OsERF67* are upregulated in submergence-tolerant genotypes in response to submergence during seed germination and coleoptile growth. Thus, both *OsERF66* and *OsERF67* are likely to have a role in submergence tolerance during seed germination in addition to their well-known role in established seedlings and mature plants. Previous studies have also shown ERF60 and ERF68 expression changes in response to submergence stress at various stages of vegetative and reproductive development [24,31].

Here, our co-expression analysis suggests that Sub1B may act as an important regulator of submergence tolerance during seed germination and coleoptile growth because it is upregulated in all five tolerant rice genotypes under submergence in comparison with the susceptible IR64 genotype (see Figure 2). Furthermore, based on promoter analysis (see Figure 4), we suggest that Sub1B may regulate OsERF66, OsERF67, OsERF61, OsHox12, OsWRKY24, OsbHLH96, OsMYB48, OsEBP89, and Os03g0622100. OsERF61 is known to show differential expression in response to abiotic stresses and is suggested to be a direct or indirect target of OsDRAP1 (Os08g0408500). OsDRAP1 is a positive regulator of drought and salinity tolerance, and OsERF61 transcript levels showed an increase in transgenic plants overexpressing OsDRAP1 [72,74]. The targets of OsERF61 are mostly other ERFs except OsCOL16, Os12g0113700, Os03g0622100, OsEBP89, OsWRKY24, OsMYB48, OsbHLH96, OsSAP1, and HSFB4A. In turn, the promoter of the OsERF61 gene has binding sites for other ERFs, OsZFP36, OSH1, OSH15, OSH71, and EREBP129 (see Figure 3 and Figure 4).

Several WRKY TFs are upregulated in rice during seed germination and coleoptile growth under submergence in tolerant genotypes, including OsWRKY1, OsWRKY8, OsWRKY16, OsWRKY24, OsWRKY31, OsWRKY70, OsWRKY71, and OsWRKY74. They are named after the WRKY domain that contains a highly conserved WRKYGQK sequence involved in binding to specific *cis*-elements in the promoters of target genes. Different WRKY TFs exhibit varying transcriptional activation/repression activities, allowing them to fine-tune the expression of target genes in response to developmental or stress-induced stimuli [40,134,135,136]. For instance, OsWRKY24 acts as both a transcriptional repressor and an activator. It contains two WRKY domains, and both are necessary for its repressor activity. OsWRKY24, OsWRKY53, and OsWRKY70 show partial functional redundancy as negative regulators of GA and ABA signaling [95,96] and rice grain size [137]. Often, WRKY TFs form dimers and heterodimers. OsWRKY71 and OsWRKY51 are induced by ABA and are highly expressed in the rice aleurone cells. These two proteins are likely to form a complex that represses GA signaling [138,139]. As shown in Figure 3 and Figure 4, multiple WRKYs bind to the same *cis*-elements in their target genes. (See Supplementary Table S2 for details on promoter binding sites.) These WRKY TFs are likely to form heterodimers of different combinations to regulate stress response and seed germination pathways. A survey of the promoter regions of *OsWRKY1*, *OsWRKY8*, *OsWRKY24*, *OsWRKY31*, *OsWRKY70*, and *OsWRKY74* suggests that OSH1 is a common regulator of all these genes. Except *OsWRKY70*, the promoters of all other WRKYs (OsWRKY1, OsWRKY8, OsWRKY16, OsWRKY24, OsWRKY31, OsWRKY71, and OsWRKY74) contain binding sites for ERFs. OsWRKY16 and OsWRKY71 promoters have binding sites only for ERFs (see Figure 3 and Figure 4). Thus, the majority of rice WRKY transcription is under the control of ERFs under submergence stress during seed germination and coleoptile elongation. However, Among all the ERFs in the group of 57 TFs, only the OsERF66 promoter contains a WRKY binding site.

Another important TF likely to be associated with submergence response is OsSAP1. It was initially identified in *O. sativa* subspecies indica as ISAP1. It is known to interact with Aminotransferase (OsAMTR1) and Pathogenesis-Related 1a Protein (OsSCP) [115] at the protein level (OsSCP and OsAMTR1 are not direct transcriptional targets of OsSAP1) [115]. *OsAMTR1* and *OsSCP* overexpressing plants showed higher seed germination, root growth, and fresh weight than wild-type plants under stress conditions. We found four abiotic stress-responsive MYB family TFs, OsMYB2, OsMYB55, OsMYB48, OsMYB21 [101,102,104,105] upregulated and one MYB TF Os05g0589400 downregulated in response to submergence during seed germination and coleoptile growth in rice. OsMYB2 and OsMYB55 positively regulate abiotic stress tolerance, possibly by promoting the synthesis of proline, arginine, soluble sugars, etc. (see Table 1 for details). None of these five MYB TFs have binding sites in the promoters of any gene included in group of 57 TFs. Therefore, if any of these genes play a role in stress response, it is more likely regulated by the ERFs.

OsbHLH6/RERJ1 acts as a positive regulator for Pi signaling and homeostasis. Overexpression of *OsbHLH6* enhances Pi accumulation [140]. It physically interacts with OsMYC2-a master regulator of JA signaling [113]. PCF8 and OsTCP18 are downregulated during seed germination under submergence stress. PCF8 is a negative regulator of cold tolerance [122]. OSTCP18 is likely to act as a transcriptional activator or repressor [123]. OsbHLH6, PCF8, and OsTCP18 do not seem to regulate other genes in the group of 57 TFs (see Figure 3 and Figure 4). Not much is known about other TFs (see Table 1).

### 2.3. Plant Reactome Knowledgebase Supports the Integration of Heterogeneous OMICs Datasets for Plant Pathways In Silico Modeling

**Figure 5:**
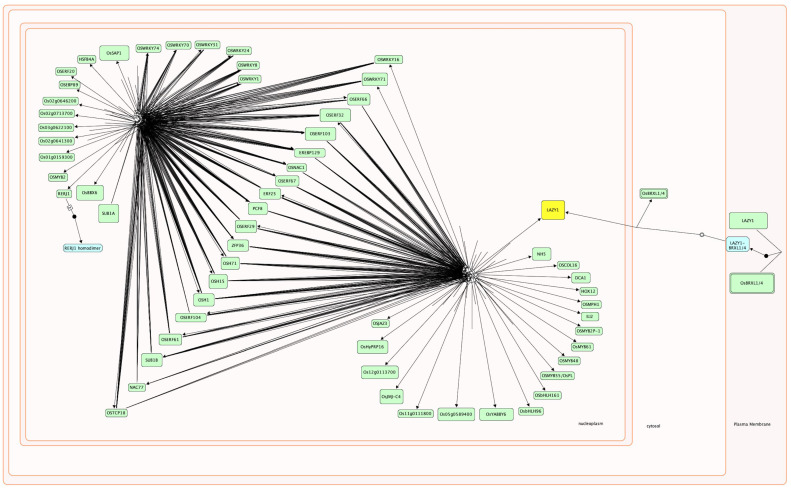
A view of a manually biocurated TF network involved in seed germination and coleoptile growth under submergence in rice (*O. sativa*) in the Plant Reactome. the *OsLAZY1*/*LA1* gene involved in shoot gravitropism is highlighted in yellow color.

We conducted a detailed literature review to improve gene functional annotation of all 57 TFs and find their association with a pathway or mechanisms. First, we collected information on gene symbols/names/synonyms, and then connected those to respective systematic RAP GeneID (see Table 1 for the many different names used in the published scientific literature for each gene). Then, we gathered information on gene/protein function, protein subcellular location, potential interactors, targets, and association with pathways or molecular mechanisms (with an appropriate citation for linking empirical evidence). After the publication of our manuscript, this information will be shared with the public databases to improve the gene annotations, as with our previous publications [1,33]. In the second step, we elucidated the connections between various TFs present within the 57 TFs. If we found experimental evidence of a direct relationship between a target and its transcription factor, we designated this as a regulatory reaction for positive or negative gene regulation. Similarly, if we identified a potential TF binding site within the promoter region of a target gene, the TF genes and it target gene share a co-expression profile, and the over-expression/under-expression of transcription factor affects the target gene’s expression; we then designated it as a positive or negative gene regulatory reaction accordingly. However, we curated a black-box reaction if the co-expression profile supports a connection, and a TF binding site is present within the promoter region of the target gene. Thus, reactions can be depicted with both high and moderate confidence. We used the Plant Reactome platform and Reactome data model to curate the transcriptional network of 57 TFs that play a key role in rice seed germination and coleoptile elongation under submergence (see Figure 5). This manually curated network identifies the TFs of central importance that coordinate and integrate developmental and environmental processes. TFs that are peripheral in this network (see Figure 5) are likely to have roles in organogenesis as discussed earlier.

We have additionally included information, when available, about protein dimerization and other regulations of TFs. For example, OsLAZY1, an important TF involved in shoot gravitropism [67,68], has two subcellular locations, the plasma membrane, and nucleus. As shown in Figure 5, OsLAZY1 interacts with BRXL4 (or BRXL1) in the plasma membrane and forms a complex that is required for subsequent nuclear localization of the OsLAZY1 [141]. OsLAZY1 acts as a TF in the nucleus, where it regulates the expression of genes involved in shoot gravitropism and tiller angle determination through negative regulation of basipetal polar auxin transport and positive regulator of lateral auxin transport. Unlike OsLAZY1, nuclear localization of its *Arabidopsis thaliana* ortholog AtLAZY1 is not essential for its function [141].

The manually curated pathway of 57 TFs involved in submergence tolerance in rice during seed germination and coleoptile growth has been submitted to the Plant Reactome database. We expect that with the upcoming database release, this pathway, and its orthology-based projections over 120 plant species will become available to the public. Users can select any of the proteins from the pathway diagram of curated or a projected pathway to see associated data on genes and proteins; visualize tissue-specific expression of specific gene projected from the EMBL-EBI Gene Expression Atlas [142]; and upload their data for analysis and visualization on Pathway Browser as described earlier [36].

## 3. Discussion

Rice fields in South Asia and Southeast Asia are regularly affected by flooding and impact ~700 million people [143]. In addition, global climate change has further intensified the need for climate-resilient crops, including flood-tolerant rice varieties. Flooding or submergence affects plants’ growth and survival at various stages (i.e., seed germination, seedling establishment, vegetative growth, flowering, and seed set), ultimately leading to yield loss [7,10,29,144]. Recent genomic and transcriptomic studies have identified major submergence tolerance quantitative trait loci (QTL) [9,144,145,146]; the major transcriptional regulators, e.g., Sub1A, SNORKEL 1, and SNORKEL 2 [11,29,30]; and several candidate genes which show differential expression in response to submergence stress in rice [1,12,24,31,32]. A major submergence tolerance QTL *Submergence 1* (*Sub1*), located on chromosome 9, was first identified from rice landrace FR13A that can endure complete submergence for two weeks or longer [29]. *Sub1* QTL consists of three genes *Sub1A*, *Sub1B,* and *Sub1C*. *Sub1A* is only present in a subset of indica accessions [29]: submergence-tolerant indica accessions possess the *Sub1A-1* allele, and submergence-sensitive indica accessions possess *Sub1A-2* [29,147]. Sub1A-1 is highly induced in response to the complete submergence of rice plants. It promotes the whole plant’s survival by inhibiting GA-induced carbohydrate consumption and elongation of the seedlings using a quiescence strategy [29,148] and supports quick recovery after de-submergence [11,149]. Many submergence-tolerant rice varieties have been developed by introgressing *Sub1A-1* [13,150]. *Sub1B* and *Sub1C* are present in all indica and japonica accessions examined to date and are not known to contribute to submergence tolerance in established plants. Sub1C induces GA-mediated carbohydrate catabolism and elongation of the seedling [147]. In some rice genotypes, *SNORKEL* 1 and *SNORKEL* 2 genes help plants to escape deep water through stem elongation and confer submergence tolerance [30]. In addition, several genes that support the anaerobic germination of rice seeds by conferring hypoxia tolerance have been identified through genome-wide association studies [9,151,152]. Thus, like most abiotic stress tolerance traits, submergence tolerance in plants, including rice, is a polygenic trait. Therefore, rice genotypes show a range of submergence tolerance. However, interactions between the rice TFs involved in seed germination have not been fully explored. Moreover, the precise mechanisms by which submergence and gravitropic signals are integrated during seed germination to activate the transcription of genes required for coleoptile elongation is still not fully understood. Understanding these mechanisms could have implications for the development of crops that are better adapted to growth in flooded environments or in the microgravity environment on space flights and ISS, and possibly beyond (e.g., the Moon and Mars).

Here we report in silico modeling of TF networks involved in seed germination and coleoptile elongation in rice under submergence using the Plant Reactome data model and platform (see Figure 5). To deduce gene–gene/gene–protein/protein–protein interactions between 57 TFs, we used evidence from gene co-expression (by re-analyzing publicly available rice transcriptome data [12]), promoter analysis, and the published literature. The TFs included in this network regulate many interconnected processes and pathways (i.e., seed germination, coleoptile growth, organ differentiation, submergence response, and gravitropism). 26 out of the 57 TFs constitute a master set of transcription factors that mediate crosstalk across multiple signaling pathways, as indicated by their binding sites in the promoter region of multiple genes within this group (see Figure 3 and Figure 4). The remaining 31 TFs that do not have binding sites within the promoters of any gene included in the group of 57 TFs seem to regulate the processes involved at the later stage of rice seedling development, including organ formation, their size, numbers (HOX12, DCA1, OsYABBY), and tiller and leaf angle formation (OsLAZY1, IL2). (See Table 1 for details.) Thus, we see the core group of TFs playing important roles during seed germination and coleoptile growth but also see the molecular signature of TFs that are preparing the ground for next stage of organogenesis and gravitropic growth (see Table 1 and Figure 5). Typically, when a plant receives an internal developmental trigger or external signal present in its immediate environment (often both), a series of molecular-genetic components are involved in signal perception, signal transduction, and transcriptional response. Most often, the focus of transcriptional data analysis is on the transcriptional response (identification of the DEG) and not on the earlier steps. We hope that considering the interactions between DEGs and their regulators may help to identify the connections when plants transition from one stage to another. Here we show the connection between genes involved in seed germination, submergence response and gravitropism in rice. This pathway and its components (like all Plant Reactome pathways) are not static and can be updated; the individual reactions and the molecular components can be linked to other reactions and pathways to support a system-level understanding of plant growth, development, and metabolism. Finally, this manually curated TF network for rice will serve as a reference for generating gene-orthology-based pathway projections for ~120 plant species. We expect that with the upcoming database release, this pathway, and orthology-based projections will become available to the public. Thus, our effort of manual biocuration of the rice pathway will also help extend knowledge about all other species hosted in the Plant Reactome knowledgebase and benefit the broader community of plant researchers. Plant Reactome provides a suite of tools for pathway visualization, analysis, and enrichment, allowing users to explore and interpret pathway models in greater detail. This provides valuable insights into the underlying molecular mechanisms and enables the generation of data-driven hypotheses for probing the complex genetic interactions in plant species of interest.

We see great potential in the re-use of publicly available genomic and transcriptomic datasets for asking new questions and mining useful information related to gene co-expression profiles/networks, and gene–gene interactions, and to understand how diverse intrinsic and extrinsic stimuli are integrated to achieve optimal growth of an organism, including plants. Moreover, by sharing and re-using existing data, researchers can save time, reduce costs, and accelerate scientific progress [153]. We recognize that manual biocuration can be a time-consuming and demanding task that requires a thorough review of published articles and OMICs data. Many researchers may not be familiar with this process. To illustrate the effort required to maintain a well-curated resource such as the pathways in the Plant Reactome and other secondary knowledgebases, we have compiled Table 1. This table includes gene summaries and mappings of synonyms and GeneIds, providing insight into the backend work that goes into maintaining these resources. By highlighting the importance of biocuration and making this information more accessible to researchers, we hope to encourage greater appreciation and use of curated databases in plant biology research. We hope this helps the younger researchers polish their literature review and data organization skills and prepare their data for submission into a public database that hosts functional information on genes or gene–gene interactions. This can be undertaken by biologists who are routinely engaged in reading papers, probing various interesting biological mechanisms, and writing reviews and manuscripts. Indeed, a community effort in biocuration can help to improve the quality and quantity of public databases and is very much needed [153,154]. Such community biocuration efforts are also needed for harmonizing and interpreting divergent datasets for knowledge synthesis. The lack of centrality in multi-data deposition, formatting, and interoperability limits the accessibility, analysis, and integration of diverse sets of omics data. Furthermore, we need genomic and pathway knowledgebases and databases that facilitate the integration of heterogenous sets of data including genomic, transcriptomic, proteomic, and metabolomic data, etc. The Plant Reactome knowledgebase provides a useful platform for integrating diverse OMICs data using a conceptual framework of system-level pathway networks. It enables visualization of plant growth, development, and metabolism, as well as plant regulation in response to both biotic and abiotic stimuli present in the plant’s immediate environment [34,39].

We hope that the Plant Reactome aids biologists in formulating data-driven hypothesis, translational research, and precision breeding. We also hope that our work inspires more researchers to engage in biocuration and contribute to the growth and improvement of public databases in this field.

## 4. Materials and Methods

### 4.1. Identification of 57 Transcription Regulators with Strong Co-Expression Pattern during Seed Germination under Submergence

We re-analyzed transcriptome data from Hsu and Tung, 2017 [12]. These data contain 2026 DEGs that are regulated in response to submergence stress in six diverse rice genotypes, including submergence-sensitive IR64 and five submergence-tolerant genotypes Nipponbare, F291, F274-2a, X8391, and X8753 [12]. IR64 is an *indica* variety, and Nipponbare is a *japonica* variety. F291 and F274-2a are recombinant inbred lines (RILs) derived from a cross between Nipponbare and IR64. The rice landrace X8391 is from Laos (IRGC 94599), and the landrace X8753 is from Indonesia (IRGC 54313) [12]. To re-analyze the data, we first updated the gene functional annotation of all 2026 DEGs and then shortlisted 183 transcription factors for further analysis. Next, we used MORPHEUS (https://software.broadinstitute.org/morpheus) to analyze the co-expression profiles of 183 transcription factor genes (see Supplementary Figure S2). Then, we selected 57 transcription factors (see Table 1) that show a strong correlation in their expression under submergence stress in tolerant genotypes compared with the IR64 susceptible genotype.

### 4.2. Promoter Analysis

A promoter analysis for all 57 transcription factor genes was conducted to identify any potential regulator and target associations within this group. Information about putative *cis*-regulatory elements located within the 1200 nucleotide sequence, including −1000 and +200 relative to the transcription start site (TSS), was extracted for all genes from the PlantPAN 3.0 database (http://plantpan.itps.ncku.edu.tw). In addition, TF-target relationships between all genes within this group were manually scored (see Supplementary Table S1).

### 4.3. Biocuration of a Transcription Factor Network

We focused on the selected 57 transcription factors involved in seed germination and coleoptile elongation under submergence and coleoptile elongation (see Table 1). For manual curation of the TF network, we first extracted the Protein ID of all 57 TFs from UniProt (https://www.uniprot.org). Next, we compiled evidence for their subcellular location from published experimental data in the literature or the CropPal database (https://crop-pal.org). Priority was given to empirical evidence over predictions. We also collected gene functional information (i.e., the phenotype of overexpression and knockout/knockdown mutants, protein–protein interaction, and gene regulation) from the literature. In the second step, gene–gene/gene–protein/protein–protein interactions (protein dimerization, protein–protein interactions, and TF–target interactions) were gathered from the literature. Using available information from literature, positive or negative correlations in the gene expression under submergence based on transcriptome data, as well as the presence of transcription activator/repressor binding site(s) within the promoter region of target gene(s), were used to synthesize the network of 57 TFs using the Reactome Curator Tool. Finally, the curated pathway was submitted to the Plant Reactome. We have previously described the manual biocuration strategy, Reactome Data Model, integration of curated pathways, and their gene-orthology-based projection into the Plant Reactome in detail [34,35,36,37].

## Data Availability

We have provided all the data in Supplementary Figures S1 and S2. The manually curated transcription factor network is submitted to the Plant Reactome (plantreactome.gramene.org), a free-of-cost database for public access.

## Supplementary Materials

The following supporting information can be downloaded at: https://www.mdpi.com/article/10.3390/plants12112146/s1. Figure S1: Sample matrix showing hierarchical clustering of 183 rice transcription factors based on their expression profile across all six rice genotypes using the MORPHEUS tool (https://software.broadinstitute.org/morpheus). The red color depicts high similarity (positive correlation), and the blue color depicts low similarity scores (negative correlation). The log10 values of the data from Hsu and Tung, 2017 [12] were used for data visualization using the MORPHEUS tool (https://software.broadinstitute.org/morpheus); Figure S2: A heatmap showing hierarchical clustering of 183 rice transcription factor genes that exhibit differential expression during seed germination and coleoptile growth under submergence. Expression levels are represented by colors, with red indicating higher expression and blue indicating lower expression. The log10 values of the data from Hsu and Tung, 2017 [12] were used for data visualization using the MORPHEUS tool (https://software.broadinstitute.org/morpheus). ‘C’ denotes controls, ‘T’ denotes samples of tolerant genotypes treated with submergence stress, and ‘TT’ is a treatment sample of the IR64 susceptible genotype. Clustering was performed among both selected genes and across different samples. Table S1: Promoter analysis of the 57 transcription factor genes used in this study.
