# Supplementary material for: Biocuration of a Transcription Factors Network Involved in Submergence Tolerance during Seed Germination and Coleoptile Elongation in Rice (Oryza sativa)
Source: Plants (Basel). 2023 May 29;12(11):2146. doi: 10.3390/plants12112146 (PMC10255735; doi:10.3390/plants12112146)
Supplement: Supplementary file 1 [file plants-12-02146-s001.zip › Figure S1 and Figure S2.pdf]

plants

MDPI

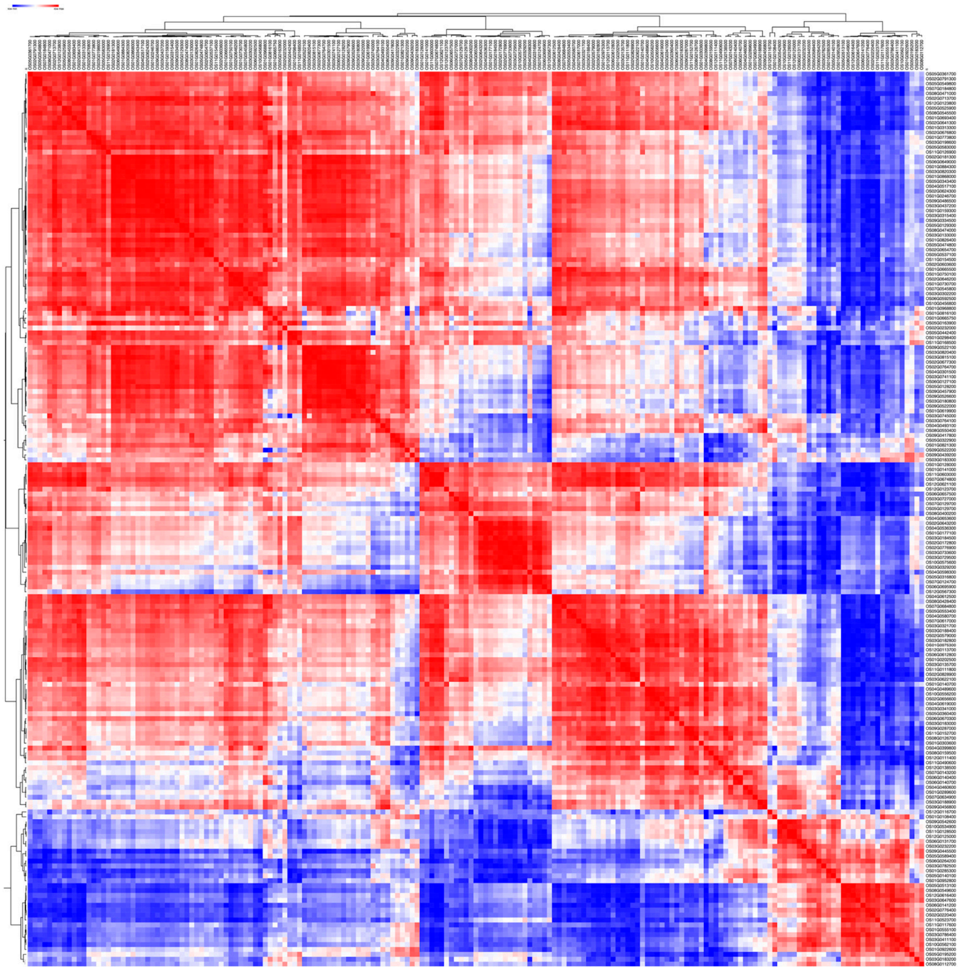

Figure S1. Sample matrix showing hierarchical clustering of 183 rice transcription factors based on their expression profile across all six rice genotypes using the MORPHEUS tool (https://software.broadinstitute.org/morpheus). The red color depicts high similarity (positive correlation), and the blue color depicts low similarity scores (negative correlation). The log10 values of the data from Hsu and Tung, 2017 [12] were used for data visualization using the MORPHEUS tool (https://software.broadinstitute.org/morpheus).

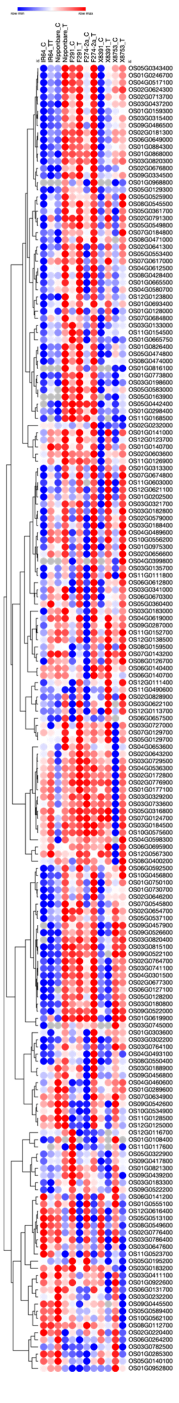

Figure S2. A heatmap showing hierarchical clustering of 183 rice transcription factor genes that exhibit differential expression during seed germination and coleoptile growth under submergence. Expression levels are represented by colors, with red indicating higher expression and blue indicating lower expression. The log10 values of the data from Hsu and Tung, 2017 [12] were used for data visualization using the MORPHEUS tool (https://software.broadinstitute.org/morpheus). 'C' denotes controls, 'T' denotes samples of tolerant genotypes treated with submergence stress, and 'TT' is a treatment sample of the IR64 susceptible genotype. Clustering was performed among both selected genes and across different samples.
